# Content Analysis of Smartphone Apps for Smoking Cessation in China: Empirical Study

**DOI:** 10.2196/mhealth.7462

**Published:** 2017-07-11

**Authors:** Feng Cheng, Junfang Xu, Chunyan Su, Xiaoxing Fu, Jonathan Bricker

**Affiliations:** ^1^ Research Center for Public Health School of Medicine Tsinghua University Beijing China; ^2^ School of Journalism and Communication China Youth University of Political Sciences Beijing China; ^3^ Institute of Anthropology Renmin University of China Beijing China; ^4^ Division of Public Health Sciences Fred Hutchinson Cancer Research Center Seattle, WA United States

**Keywords:** smoking cessation, smartphone apps, China

## Abstract

**Background:**

With 360 million smokers, China consumes more cigarettes than any other country in the world. Given that 620 million Chinese own smartphones, smartphone apps for smoking cessation are increasingly used in China to help smokers quit.

**Objective:**

This study analyzed and evaluated the contents of all smoking cessation apps (iOS and Android) available in China, applying the China Clinical Smoking Cessation Guideline (CCSCG; identical to the US Clinical Practice Guideline for Treating Tobacco Use and Dependence) as a framework for analysis.

**Methods:**

We conducted a content analysis of Chinese Android and iOS smoking cessation apps (N=64) designed to assist users in quitting smoking. Each app was independently coded by two raters for its approach to smoking cessation and adherence to the CCSCG. We also recorded the features of smoking cessation apps (eg, release date, size, frequency of downloads, user ratings, type, quality scores by raters, and designers). Linear regression was used to test predictors of popularity and user-rated quality.

**Results:**

Chinese smoking cessation apps have low levels of adherence to guidelines, with an average score of 11.1 for Android and 14.6 for iOS apps on a scale of 0 to 46. There was no significant association between popularity, user rating, and the characteristics of apps. However, there was a positive relationship between popularity, user rating, and adherence score.

**Conclusions:**

Chinese apps for smoking cessation have low levels of adherence to standard clinical practice guidelines. New apps need be developed and existing apps be revised following evidence-based principles in China.

## Introduction

Tobacco use is one of the most serious public health problems in the world. About 6 million people die every year from tobacco use, and this number is projected to reach about 1 billion by 2030 [1,2]. China has the largest population of smokers in the world, with an estimate 360 million smokers in 2015 [3]. Moreover, the per capita number of cigarettes smoked in China has reached 15.2 per day [3]. In China, an astounding 68% of men smoke, as compared to 3.2% of women [4]. Cigarette smoking leads 1 million Chinese people to die each year. While China is starting to adopt smoke-free policies and open cessation clinics, if no further tobacco control measures were adopt by the Chinese government, 2 million people in China will die from smoking per year in 2030 and this figure will increase to 3 million by the year 2050, contributing greatly to the Chinese burden of disease [4].

The Chinese government and the community have carried out extensive publicity and education in tobacco control [5-8]. Many evidence-based smoking cessation methods (eg, psychological and behavioral intervention, telephone intervention therapy, and acupuncture therapy) have been implemented to assist in smoking cessation [9-12]. To promote smoking cessation for smokers, the first version of the China Clinical Smoking Cessation Guideline (CCSCG), modeled directly from the US Clinical Practice Guideline for Treating Tobacco Use and Dependence [13], was issued in 2007 and updated in 2015 and contains empirically supported strategies and recommendations designed to assist health care providers in the delivery of effective treatments for tobacco cessation. These include pharmacological interventions (eg, nicotine replacement therapy) and behavioral interventions (eg, motivational messaging, making a quit plan) [14]. While these approaches are valuable, new approaches with high reach and low cost are needed to control tobacco use on a broad scale. Smartphones may offer one such new approach. About 50% of the Chinese population, which is roughly 620 million people, own smartphones [15]. Because of the portability of smartphones, which enable access 24 hours a day, long-term management and reinforcement of health behaviors via smartphone apps has promise [16-20].

In recent years, smartphone app interventions have been increasingly used as platforms for health promotion including facilitating smoking cessation [21,22], providing diabetes education [23], encouraging attendance of primary care appointments [24], and even encouraging sunscreen application [25]. For smokers, smoking cessation is the single most important change they can make to their behavior to improve their life expectancy and quality of life [26-27]. Currently, several studies have evaluated the content of smoking cessation apps in high-income countries especially in America. A recent study found that smoking cessation apps were downloaded more than 700,000 times every month [28]. In another study, almost half of smokers had used an app to support their quit attempt [29]. If such apps support behavior change, they could confer a considerable benefit to public health considering the significant risks of smoking [30]. Indeed, current evidence has demonstrated the usefulness of mobile technology in supporting smoking cessation [24,25,31].

In China, there were approximately 137 million health and fitness app users in 2015 [15]. A variety of smartphone apps for smoking cessation were released between 2009 and 2016 in China. About 70 apps are aimed at helping smokers quit in China, but very little is known about their content, which is important for determining their potential value for smoking cessation. To address this issue, this study analyzed and evaluated the contents of all smoking cessation apps (iOS & Android) available in China, applying the CCSCG as a framework for analysis [14,32].

## Methods

### Data Sources

A total of 101 relevant iPhone apps were identified by searching for keywords “quit smoking,” “stop smoking,” and “smoking cessation” in Mandarin Chinese in the iPhone app store in February 2016. A total of 74 apps—53 irrelevant apps (eg, tobacco knowledge apps, male health apps and medical health apps), 5 duplicate apps, 2 apps which could not be opened, and 14 English language apps—were excluded by checking the content after download. The remaining 27 iPhone smoking cessation apps, including 26 free and 1 paid app, were evaluated for the study.

**Figure 1:**
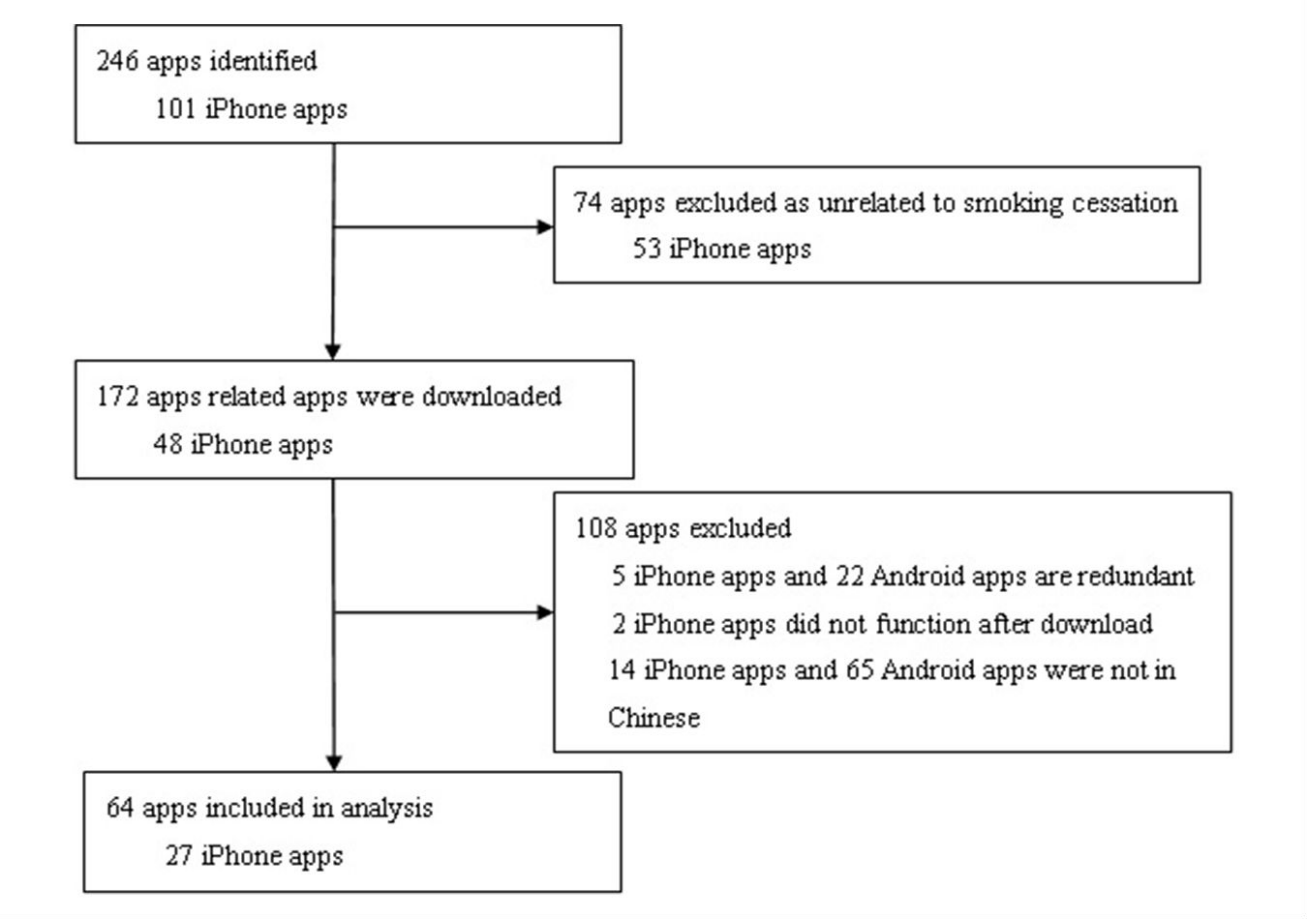
Procedure for selecting Chinese smoking cessation apps.

A total of 145 Android apps were identified from the 360 Assistant app store (86 apps) and Baidu Assistant app store (59 apps), which account for 87.4% of all Android app downloads in China, by searching for the keywords “quit smoking,” “stop smoking,” and “smoking cessation” in Mandarin Chinese in February 2016. A total of 108 apps—21 irrelevant apps (eg, tobacco advertisement apps, expertise in the tobacco industry apps), 15 duplicate apps, 7 prior version apps, and 65 English language apps—were excluded by checking the content after download. The remaining 37 free Android smoking cessation apps were evaluated for this study. In total, the analytical sample included 64 apps (Figure 1).

### Coding of Smoking Cessation Apps

For each app, descriptive information was retrieved regarding its characteristics (ie, release date, size, type), popularity (frequency of downloads), and user rating (ie, app was rated ranging from 1 to 5 stars) (Table 1). Apps were categorized into different types: (1) calculators, that calculate number of days, amount of money saved, health improvements since quitting; (2) calendars, that track number of cigarettes per day, smoking pattern, and mood; (3) hypnosis, that use hypnosis techniques for smoking cessation; (4) rationing, that ration the number of cigarettes for a specific time period; and (5) other, for apps that did not fall into the previous categories or fell into multiple categories.

Apps were also coded for their level of adherence to the CCSCG [14]. To measure adherence to the CCSCG, an index of 23 items was developed that closely followed the guidelines. Each app was independently coded by two reviewers on each of the 23 items in the adherence index using a score ranging from 0 to 2. A score of 2 indicated that the feature was fully present, a score of 1 indicated that the feature was partially present, and a score of 0 indicated that it was not present at all. For example, for the guideline to recommend the use of medicine, apps that did not mention medicine received a score of 0, apps that partially mentioned medicine received a score of 1, and a clear and strong mention (fully presented the specific drug, proper use, dosing, and common side effects) for medicine received a score of 2.

A total of 87% (20/23) of items were ratings of concrete features, which by their very nature did not lend themselves to rater judgment and interpretation. For example, the Ask criterion measures whether or not the app asks the user if he or she smokes, and if so, how many cigarettes per day. The concrete quality of these ratings was reflected in the fact that there was 100% interrater agreement for these 20 items.

In 13% (3/23) of items, the guidelines were subjective (eg, presence of practical advice for quitting smoking). For these 3 items, there was a small fraction of interrater disagreement: 23% of the time for the iPhone apps and 15% for the Android apps. In these cases, we minimized subjectivity of rating with two coders discussing and resolved disagreement when the score differed by 1 (19% for iPhone and 8% for Android) and averaged the scores. When the score differed by 2 (5% for iPhone and 1% for Android), a gold standard outside coder resolved score discrepancies 100% of the time. The maximum possible score on the index was 46 for each app.

### Statistical Analysis

The statistical analysis was performed using SPSS version 20.0 software (IBM Corp). Total *n*, percentages, mean, and standard deviation were calculated to describe the features of apps. Weighted Cohen’s kappa and intraclass correlation coefficient were used to evaluate interrater agreement. In addition, to identify features of the apps which were associated with the popularity and user-rated quality, linear regression was used to test predictors of popularity (frequency of download) and user-rated quality (user rating with number of stars). Analyses regarding the user-rated quality of the apps were restricted to the apps that had star ratings (73% of the apps), and analyses regarding the popularity of apps were limited to the Android apps because iOS does not release data on the frequency of downloads for iPhone apps. The sample of 64 had 99% power to observe an effect size of 36.8% for the analysis of predictors of quality and downloads.

**Table 1:** Characteristics of Chinese smoking cessation apps.

Characteristic		Amount
**Release date, n (%)**		
	2009	2 (3)
	2010	1 (2)
	2011	6 (9)
	2012	8 (13)
	2013	8 (13)
	2014	12 (19)
	2015	6 (9)
	2016	1 (2)
	Unknown	20 (31)
Average file size (in megabytes), mean (SD)		11.05 (14.28)
**Frequency of downloads^a^, n (%)**		
	10-100	4 (11)
	100-1000	12 (33)
	1000-10,000	14 (39)
	>10,000	6 (17)
**User ratings, n (%)**		
	1 star	2 (3)
	2 stars	6 (9)
	3 stars	28 (44)
	4 stars	8 (13)
	5 stars	3 (5)
	Missing	17 (27)
**Function^b^, n (%)**		
	Calculator	34 (53)
	Calendar	27 (42)
	Hypnosis	1 (2)
	Rationing	8 (13)
	Other	21 (33)
**Designers, n (%)**		
	Individuals or technology companies	58 (91)
	Government agencies	3 (0)
	Unidentified sources	3 (0)

^a^Android apps only.

^b^A total of 42% of apps had multiple functions.

## Results

### Basic Features

The characteristics of the 64 smoking cessation apps included in the analysis are presented in Table 1. Smartphone apps for smoking cessation started to appear in China in 2009. The largest number of app releases (12, 19%) was in the year 2010. The average size for all apps in the sample was 11.05 MB, and the frequency of download for 83% of apps was less than 10,000 times. The modal user rating for most apps (44%) was 3 or 4 stars (out of 5). Most apps used a calculator approach (53%), followed by calendar (42%), other (33%), rationing (13%), and hypnosis (2%). Most apps (91%) were designed and developed by individuals or technology companies rather than government agencies.

### Adherence Scores

With a maximum score on the index of 46, the average score was 14.6 for iPhone apps and 11.1 for Android apps (Multimedia Appendix 1). For iPhone, the Quit Smoking at Once app had the highest score of 38, while apps 30 Day Messages for Quitting and Common Medical Knowledge received the lowest score of 2.5. For Android, the Quit Smoking App had the highest score of 25, while the apps 30 Day Quit Message, Quitting App, and Self-Awareness of Smoking Habits had the lowest score of 3.

### Influencing Factors

There was no significant association between popularity, user rating, and the characteristics of apps (*P*>.05). However, there was a positive relationship between popularity, user rating, and adherence score as seen in Table 2.

**Table 2:** Regression analysis of app characteristics on app popularity and user-rated quality .

App characteristics	Popularity		User-rated quality	
	Beta	*P* value	Beta	*P* value
Release date	0.180	.639	–0.033	.934
Average file size	0.024	.953	–0.255	.537
Type	0.375	.354	0.116	.786
Frequency of downloads	—	—	0.123	.712
User rating	0.116	.712	—	—
Adherence score	0.323	.398	0.090	.823

## Discussion

### Principal Findings

This was the first study to analyze the content of all publicly available smoking cessation apps in China. The results showed that Chinese apps generally lack the content that is consistent with the CCSCG [14]. The number of existing smoking cessation apps in China is smaller than that of America because of the poorer development of eHealth interventions in China [33]. However, the adherence score of Chinese apps to the CCSCG is consistent with analyses of English apps (average adherence scores for iPhone and Android are 14.2 and 11.7, respectively, on 0 to 42 score) [33,34]. One reason may be that the developers of Chinese and English apps are not aware of or do not understand the importance of clinical practice guidelines, as most designers of smoking cessation apps are individuals or companies without any background in clinical practice for tobacco cessation. Indeed, the adherence of smoking cessation apps designed by government agencies was higher than that of other designers in the study.

The results showed that the content of Chinese apps is primarily limited to calculators (eg, money saved by not smoking) and calendars (eg, showing smoking patterns over time). While such tracking-derived information is potentially useful, it vastly underutilizes the potential of smartphone apps to provide evidence-based advice, motivation, and training in skills to cope with cravings and recover from relapses. In addition, the sensors (eg, Global Positioning System) already built into most smartphones make it possible for smartphone apps to provide sophisticated just-in-time and in-the-moment intervention for smoking cessation. In sum, the content of current Chinese apps was generally simplistic, and the potential for improvement is enormous.

Compared to the large population of smokers and smartphone owners, Chinese language smoking cessation apps had relatively few downloads. Indeed, only 6 Android apps in Chinese had more than 10,000 total downloads. However, 35.2% of US smoking cessation apps had more than 10,000 downloads with 7.8% having more than 100,000 downloads [33]. This disparity may be due to Chinese smokers’ lack of awareness of smoking cessation apps and smoking cessation advice in general. In traditional Chinese culture, smoking functions as a way to make social connections, smooth social interactions, and express one’s social and economic position. Furthermore, in the comments on smoking cessation apps, many users said that stopping smoking was very easy and they will be lucky to avoid the health consequences of smoking, which implies they do not want or understand the value of smoking cessation assistance [35]. Once they download an app, users appear to respond positively to it if it follows the CCSCG, as the adherence score was positively related to app popularity and user-used quality. However, more research is needed to understand what Chinese smokers know about smoking cessation advice, their interest in receiving it, and methods to overcome barriers they may have to using it. When smoking cessation apps that follow the CCSCG are developed that are appropriate and motivating to Chinese smokers, it would be highly valuable to test their efficacy. Indeed, the need for efficacy data is underscored by the fact that to date only randomized trials of smoking cessation apps developed in English have been published [36]. When Chinese apps show promise in randomized trials, public health media campaigns to widely publicize their existence and potential value for smokers would serve the role of promoting the adoption of proven interventions on a broad scale.

An important future research question is how to effectively reach a general population of smokers. A recent US survey of smokers showed that 91% had experience using smartphone apps [37], which suggests that a smoking cessation app in the United States could potentially reach users with demographics that are generally representative of the overall population of US smokers. Research is now needed to determine what fraction of the Chinese smokers have experience using apps and would be interested in using one to quit smoking, with the aim of determining demographic characteristics of the target population for quit smoking apps in China. As the purchase of iPhone and Android smartphones is rising among old and young people with lower socioeconomic status in China [38], the potential utility of smoking cessation apps for Chinese smokers will grow.

The results of the current studies in the United States and China indicate that despite the recent expansion of smartphone platforms and increased availability of apps for cessation, existing apps still lack many elements that are generally recommended for quitting smoking. We recommend that future research integrate the content of the clinical practice guidelines that have been proven effective in randomized clinical trails [39-41]. Once guidelines are integrated into to the content of Chinese language smartphone apps, these apps can be tested in randomized trials of Chinese smokers.

The proliferation of smartphones can now make them valuable health promotion tools. This study presents an evidence-based evaluation of a tool (smoking cessation apps) for health promotion in China that could inform health policy makers of an eHealth intervention with promise for widespread public health impact. As these apps proliferate in China, there is a great potential that data scientists can analyze large databases of users to understand patterns of use to inform personal tailoring of app content to specific users.

### Limitations

This study has limitations. First, like other content analysis, the data were rated by trained raters. Our method for minimizing rater bias was having 87% of all items be of concrete criterion with the remaining 13% having a small fraction of disagreement that was resolved 100% of the time via a rigorous process described in the methods. Second, the coding measured the presence or absence of the clinical guideline, not the degree to which that guideline was followed or presented effectively. Finally, not all app users provided quality ratings. In the study, an app’s number of stars was used as an indicator of its user-perceived quality, so the generalizability of those results is unknown.

### Conclusions

Chinese apps for smoking cessation lack content that is known to be effective for smoking cessation. Including content based on China’s clinical practice guidelines would be extremely valuable for improving existing apps and informing the development of new apps and thereby increasing the chances they will make a significant impact on the smoking epidemic in China.

## Appendix

Multimedia Appendix 1 The adherence score of Chinese apps to the China Clinical Smoking Cessation Guideline.

## Abbreviations

CCSCG: China Clinical Smoking Cessation Guideline
