# Supplementary material for: Content Analysis of Smartphone Apps for Smoking Cessation in China: Empirical Study
Source: JMIR Mhealth Uhealth. 2017 Jul 11;5(7):e93. doi: 10.2196/mhealth.7462 (PMC5589410; doi:10.2196/mhealth.7462)
Supplement: Multimedia Appendix 1 [file mhealth_v5i7e93_app1.pdf]

Multimedia Appendix 1. The adherence score of Chinese apps to the China Clinical Smoking Cessation Guideline

| Android | | iPhone | |
|---|---|---|---|
| App number | Adherence Score | iPhone number | Adherence Score |
| a01 | 10.0 | i01 | 24.5 |
| a02 | 3.0 | i02 | 2.5 |
| a03 | 7.0 | i03 | 15.5 |
| a04 | 12.0 | i04 | 14.0 |
| a05 | 12.0 | i05 | 16.0 |
| a06 | 7.0 | i06 | 6.0 |
| a07 | 10.0 | i07 | 38.0 |
| a08 | 9.0 | i08 | 4.0 |
| a09 | 11.0 | i09 | 20.0 |
| a10 | 12.0 | i10 | 7.0 |
| a11 | 15.0 | i11 | 15.5 |
| a12 | 22.0 | i12 | 18.0 |
| a13 | 4.0 | i13 | 16.5 |
| a14 | 25.0 | i14 | 17.0 |
| a15 | 5.0 | i15 | 13.0 |
| a16 | 19.0 | i16 | 18.5 |
| a17 | 22.0 | i17 | 15.0 |
| a18 | 12.0 | i18 | 15.0 |
| a19 | 3.0 | i19 | 9.5 |
| a20 | 16.0 | i20 | 17.5 |
| a21 | 5.0 | i21 | 9.5 |
| a22 | 11.0 | i22 | 29.5 |
| a23 | 12.0 | i23 | 2.5 |
| a24 | 14.0 | i24 | 6.0 |
| a25 | 16.0 | i25 | 3.0 |
| a26 | 12.0 | i26 | 4.0 |
| a27 | 6.0 | i27 | 36.5 |
| a28 | 15.0 | Average score | 14.6 |
| a29 | 12.0 | | |
| a30 | 10.0 | | |
| a31 | 7.0 | | |
| a32 | 14.0 | | |
| a33 | 11.0 | | |
| a34 | 12.0 | | |
| a35 | 3.0 | | |
| a36 | 10.0 | | |
| a37 | 5.0 | | |
| Average score | 11.1 | | |
